# Is a Single Lipoprotein(a) Measurement Once in a Lifetime Sufficient? The Results from the STAR-Lp(a) Study †

**DOI:** 10.3390/medsci13040320

**Published:** 2025-12-15

**Authors:** Monika Burzyńska, Piotr Jankowski, Maciej Banach, Michał Chudzik

**Affiliations:** 1 Department of Epidemiology and Biostatistics, Medical University of Lodz, 90-752 Lodz, Poland; 2 Department of Internal Medicine and Geriatric Cardiology, Centre of Postgraduate Medical Education, 01-813 Warszawa, Poland; 3 Department of Epidemiology and Health Promotion, School of Public Health, Centre of Postgraduate Medical Education, 01-826 Warszawa, Poland; 4 Department of Preventive Cardiology and Lipidology, Medical University of Lodz, 93-338 Lodz, Poland; 5 Ciccarone Center for the Prevention of Cardiovascular Disease, Johns Hopkins University School of Medicine, Baltimore, 21287 MD, USA; 6 Faculty of Medicine, The John Paul II Catholic University of Lublin, 20-708 Lublin, Poland; 7 Nephrology, Hypertension and Family Medicine Department, Medical University of Lodz, 90-549 Lodz, Poland

**Keywords:** lipoprotein(a), cardiovascular risk, lipid profile

## Abstract

**Background:** Guidelines suggest that a single lifetime measurement of lipoproteina(a) [Lp(a)] is sufficient for most patients as its levels are largely genetically determined and do not significantly change over time. The aim of the study was to assess the midterm variability in Lp(a) levels and its determinants. **Methods:** The analysis included 1263 patients (68.7% women, median age 69.0 [59.0–75.0] years) who underwent two measurements of Lp(a) levels at an interval of at least one year and up to a maximum of three years. **Results:** The median Lp(a) level in the first measurement was 9.0 ± 19.0 mg/dl, compared to 8.8 ± 19.1 mg/dL in the second measurement (*p* < 0.001). The mean increase in Lp(a) level (N = 692) was 4.1 ± 6.9 mg/dL, while the mean decrease (N = 483) was 5.6 ± 11.4 mg/dL. A total of 64.7% of patients exhibited a change in Lp(a) level ≥ 10%, 44.3% ≥ 20%; 28.2% ≥ 30% and 14.0% ≥ 50% of the baseline values. We found no significant differences in the Lp(a) level change related to sex, age, or comorbidities. **Conclusions:** A significant change in Lp(a) levels was observed in the midterm follow-up. These findings potentially have a profound clinical importance. The current expert recommendation to measure Lp(a) at least once in a lifetime appears to be inaccurate and should be revised.

## 1. Introduction

Lipoprotein(a) [Lp(a)] is a lipoprotein structurally similar to low-density lipoprotein (LDL), with the addition of a unique apolipoprotein(a) moiety [1,2]. It was first described in 1963 by Berg, who identified it as an antigen-like material in rabbits immunized with human low-density lipoprotein. Structurally, Lp(a) resembles an LDL particle, consisting of a cholesteryl ester-rich lipid core and a single molecule of apolipoprotein B-100 (apoB). What distinguishes Lp(a) is the covalent attachment of apoB to apolipoprotein(a) [apo(a)], a liver-derived glycoprotein that confers the particle’s unique properties. Apo(a) contains multiple repeating sequences organized into characteristic triple-loop motifs known as kringles (K), which share structural homology with domains of the plasminogen gene [3].

For several decades, elevated Lp(a) concentrations have been recognized as an independent risk factor for coronary artery disease, ischemic stroke, and aortic valve stenosis [4]. Large meta-analyses have consistently demonstrated that cardiovascular risk increases in a linear fashion with rising Lp(a) levels [5]. Because Lp(a) concentrations are largely genetically determined, current clinical guidelines recommend a single lifetime measurement of Lp(a), based on the long-standing assumption that its levels remain stable over time [6,7,8,9]. However, a growing body of evidence suggests that Lp(a) concentrations may vary under the influence of hormonal, metabolic, inflammatory and renal factors [10,11]. Such variability may have important clinical implications: a single measurement obtained during a period of transient disturbances may not accurately reflect a patient’s long-term risk profile. Most patients at high cardiovascular risk, including those with elevated Lp(a), are managed in outpatient cardiology clinics. Although several studies have examined the prevalence of elevated Lp(a) levels [12,13,14,15], they primarily focused on cross-sectional assessments and did not evaluate the variability of Lp(a) over time. Furthermore, little is known about the factors that may influence fluctuations in Lp(a) levels. Addressing this gap, the present study aimed to investigate the midterm variability in Lp(a) concentrations and to identify determinants of these changes in consecutive outpatient patients.

## 2. Materials and Methods

We analyzed data of the SpecialisT cARe (STAR)-Lp(a) cohort study participants. We prospectively enrolled consecutive patients referred to two outpatient cardiology clinics between March 2022 and March 2025. The analysis included 1263 patients who underwent two Lp(a) measurements performed at intervals of at least one year and up to a maximum of three years. Lp(a) concentrations were determined using an enzyme-linked immunosorbent assay (ELISA; Mercodia Lp(a) ELISA, Cat. No. 10-1016-01, Mercodia AB, Uppsala, Sweden). The assay employed a two-site sandwich ELISA with monoclonal antibodies directed against isoform-insensitive epitopes on apolipoprotein(a), minimizing variability related to kringle IV type 2 repeat heterogeneity. Calibration was performed with a purified Lp(a) standard traceable to WHO/IFCC reference material (SRM 2B), ensuring metrological traceability and inter-laboratory comparability. The assay’s native reporting units were mg/dL, with a reportable range of 0.3–90 mg/dL. Intra- and inter-assay coefficients of variation were <6% across the analytical range, confirming high reproducibility. In accordance with current recommendations, results are reported in the assay’s native units (mg/dL). Venous blood was collected in the morning after an overnight fast, processed within two hours of collection and analyzed immediately. To place participants into clinically meaningful categories, Lp(a) concentrations were classified into three risk groups commonly used in the literature and endorsed by expert statements: <30 mg/dL (low risk); 30–50 mg/dL (intermediate risk) and ≥50 mg/dL (high risk) [16].

The following quantitative variables were included in the analysis: age, body mass index (BMI), total cholesterol (TC), low-density lipoprotein cholesterol (LDL-C), high-density lipoprotein cholesterol (HDL-C), triglycerides (TG), hemoglobin A1c (Hb_A1c_), homocysteine, and C-reactive protein (CRP). Qualitative variables included sex and the presence of comorbidities, namely hypertension, hyperlipidemia, diabetes, asthma, rheumatoid arthritis (RA), migraine, thrombophlebitis, stroke, and myocardial infarction. Smoking status was also considered. Comorbidities were defined according to the current guidelines [17,18,19,20]. The study was approved by the Bioethics Committee of the Lodz Regional Medical Chamber (K.B.-0115/2021, Approval date: 7 July 2021). Informed consent was obtained from all subjects involved in the study.

### Statistical Analysis

Structure indices were calculated for qualitative variables. Associations between qualitative variables were assessed using the χ^2^ test. Quantitative variables were analyzed using descriptive statistics (mean, standard deviation [SD], median, interquartile range [IQR], minimum, maximum). The Shapiro–Wilk test was applied to assess the normality of distribution. For quantitative variables, the nonparametric Mann–Whitney U test, the Kruskal–Wallis test or test for differences between groups was used, as appropriate. Spearman’s correlation coefficient was calculated to assess correlations between quantitative variables. To identify factors independently associated with elevated Lp(a) levels, a stepwise backward multivariable logistic regression was performed. The Bonferroni-corrected *p* value (<0.003) was used to account for multiple comparisons. Statistical analyses were performed using Statistica version 13.1 (TIBCO Software Inc. Palo Alto, CA, United States).

## 3. Results

A total of 1263 participants (394 men and 869 women) were included in the study (Table 1).

**Figure 1:**
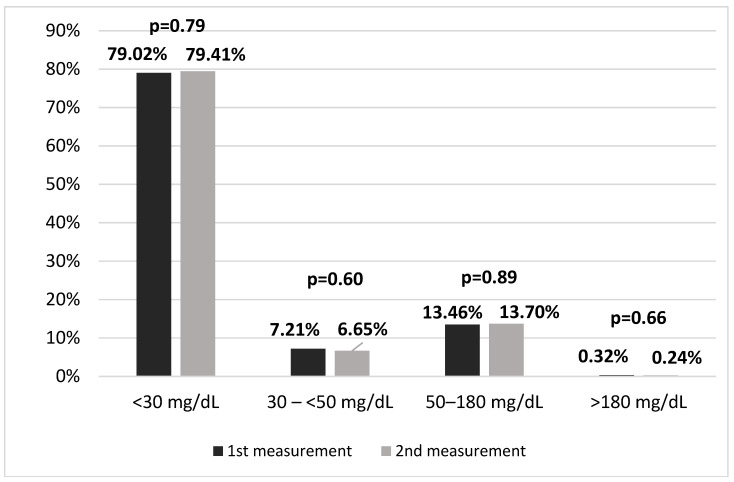
The proportions of patients according to the Lp(a) concentration category at the baseline and follow-up measurements.

**Figure 2:**
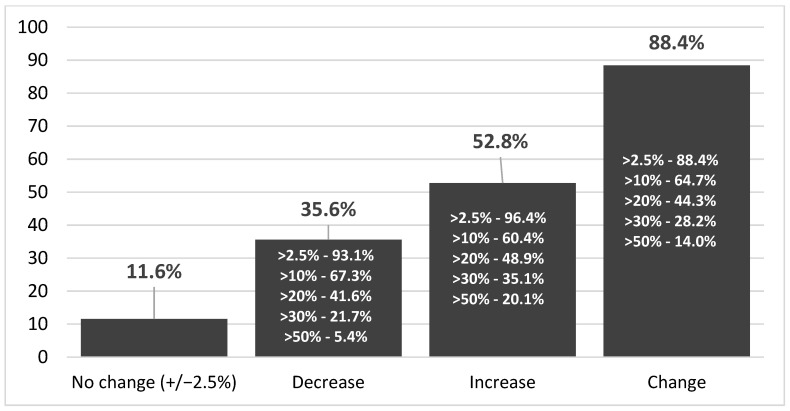
The proportions of patients according to the Lp(a) concentration change between 1st and 2nd measurements.

**Figure 3:**
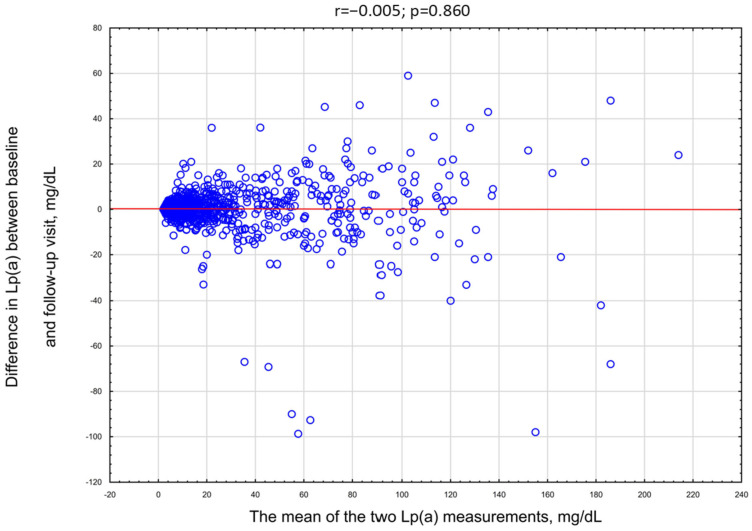
The correlation between the mean of the two Lp(a) measurements and the difference between 1st and 2nd Lp(a) measurements.

The median age was 69 years (59–75 years). The age range of the included subjects was between 21.0 and 96.0 years. The median time between the first and second measurement was 12 months. There was no significant correlation between follow-up length and absolute or relative change in Lp(a) concentration (r = 0.03, *p* = 0.67). The mean Lp(a) concentration at the first measurement was 21.7 (SD 30.7) mg/dL, with a median of 9.0 (IQR 4.0–23.0) mg/dL. At the second measurement, the mean was 21.9 (SD 30.6) mg/dL, with a median of 8.8 (IQR 4.9–24.0) mg/dL (*p* < 0.001). Figure 1 illustrates the distribution of patients across Lp(a) categories at both time points. The figure summarizes group-level categories rather than individual trajectories. Although the group-level means were nearly identical, there was substantial individual variability. The mean percentage change between the two measurements was 36.2% (SD 288.0%), with a median of 16.7% (IQR 6.5–33.3%) (Table 2). In Figure 2, we present the distribution of participants across progressively higher thresholds of relative Lp(a) change. It shows the proportion of participants who met at least each respective level of change. In the total cohort, 88.4% of individuals showed a change of at least ±2.5%. The mean increase in Lp(a) level (N = 692) was 4.1 ± 6.9 mg/dL, while the mean decrease (N = 483) was 5.6 ± 11.4 mg/dL. The difference between 1st and 2nd Lp(a) measurement was not correlated with the mean of the two measurements (Figure 3).

**Figure 4:**
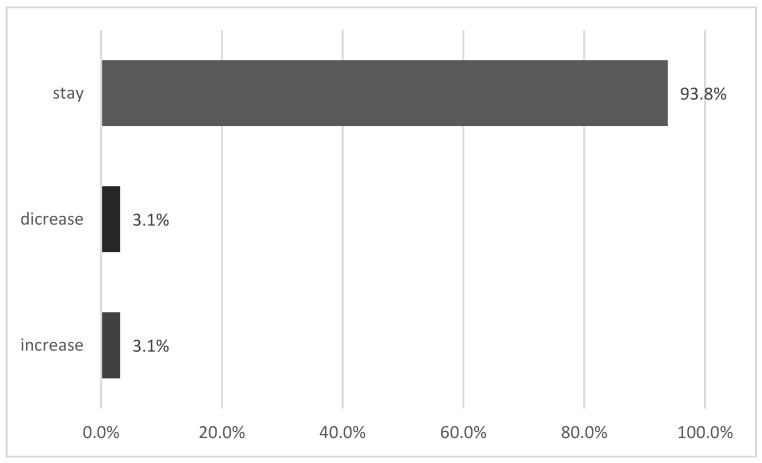
Change risk category based on Lp(a) level.

The analysis of changes in Lp(a)-based risk categories between the first and second measurement demonstrated a predominantly stable classification profile, yet it also revealed a measurable proportion of clinically relevant reclassification. In most participants (93.8%), the risk category remained unchanged across the two assessments, confirming the generally stable nature of Lp(a) concentrations over time (Figure 4).

However, a non-negligible subset of patients (6.2%) did experience risk category shifts, with 3.1% moving to a lower and 3.1% to a higher category. Although these proportions are relatively small, they indicate that Lp(a) is not entirely immutable and that individual variability does occur, potentially reflecting biological fluctuations or changes in measurement precision.

These observations suggest that while Lp(a) is overall a stable biomarker, periodic reassessment may still be warranted, particularly in patients near clinical decision thresholds or in those with evolving cardiovascular risk profiles.

When stratified according to the degree of change in Lp(a) concentrations between baseline and follow-up, 703 individuals (55.7%) demonstrated a change of <20%, whereas 560 participants (44.3%) had a change of ≥20% (Table 1). The distribution of sex and age was similar between groups.

**Figure 5:**
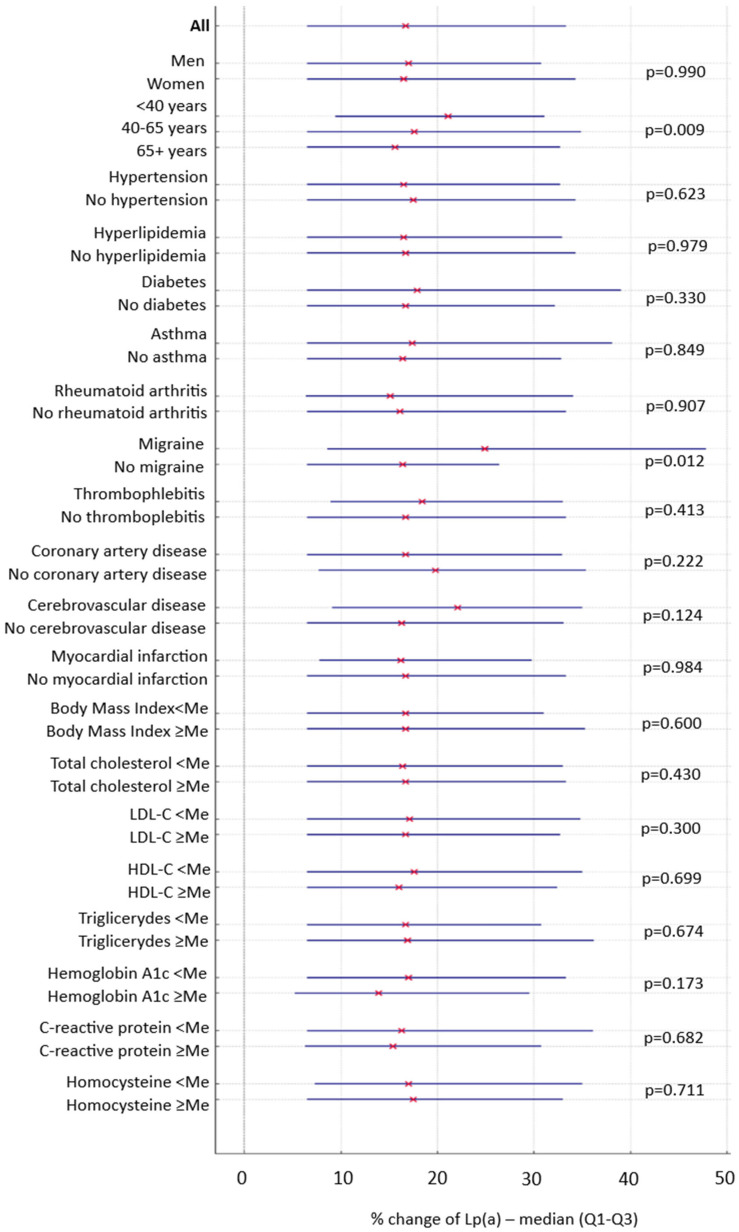
Percentage change in Lp(a) concentrations between the two measurements across subgroups. “x” stand for medians, while whiskers stand for interquartile ranges.

The distribution of percentage change across predefined subgroups is shown in Figure 5. Median percentage changes (with interquartile ranges) were comparable between men (17.0%, IQR 6.5–30.8) and women (16.5%, IQR 6.5–34.3, *p* = 0.990). Similarly, no significant differences were observed between individuals with and without hypertension (16.5%, IQR 6.5–32.7 vs. 17.5%, IQR 6.5–34.3; *p* = 0.623), hyperlipidemia (16.5%, IQR 6.5–32.9 vs. 16.7%, IQR 6.5–34.3; *p* = 0.979), diabetes (17.9%, IQR 6.5–39.0 vs. 16.7%, IQR 6.5–32.2; *p* = 0.330), coronary artery disease (16.7%, IQR 6.5–32.9 vs. 19.8%, IQR 7.7–35.4; *p* = 0.222) asthma (17.4%, IQR 6.5–38.1 vs. 16.4%, IQR 6.5–32.8; *p* = 0.849), or rheumatoid arthritis (15.1%, IQR 6.4–34.1 vs. 16.1%, IQR 6.5–33.3; *p* = 0.907).

Greater changes were observed in some subgroups. Participants younger than 40 years tended to have a significantly larger median change (21.1%, IQR 9.4–31.1) compared with those aged 40–65 (17.6%, IQR 6.5–34.9) and ≥65 years (15.6%, IQR 6.5–32.7, *p* = 0.009). Individuals with a history of migraine also tended to have higher variability (24.9%, IQR 8.6–47.8) compared with those without migraine (16.4%, IQR 6.5–26.4, *p* = 0.012). No factors independently associated with the percentage change in Lp(a) between the two measurements were identified in the stepwise backward multivariable logistic regression model.

## 4. Discussion

Lipoprotein(a) is a distinct lipoprotein with atherothrombogenic properties. Although its plasma concentration is largely genetically determined, several factors may contribute to variability, which in turn can affect its clinical interpretation and utility. The STAR-Lp(a) study provides important new evidence that Lp(a) levels are not invariably stable over time in clinically stable outpatient patients. Nearly half of the participants demonstrated a change of ≥20% between two consecutive measurements, and the median intra-individual variability was 16.7%. These findings directly challenge the prevailing assumption that a single lifetime measurement of Lp(a) is sufficient for risk stratification. Indeed, the present results suggest that the current recommendations of a single lifetime measurement of Lp(a) may be inadequate. Indeed, reliance on a single measurement may lead to inaccurate or misleading clinical decisions. On the other hand, since the mean and median values of the first and second measurements were very similar, it appears that a single Lp(a) measurement is sufficient for epidemiological studies.

Our results are consistent with recent studies documenting substantial variability in Lp(a) concentrations across different populations. In the Nashville cohort, 40.5% of individuals experienced a change of ≥25% and 38.1% had an absolute difference of at least 10 mg/dL between repeat assessments, despite being clinically stable. Greater variability was observed in Black individuals compared with White individuals, and in women compared with men [21]. In the study by Deshotels et al., black race and female sex were significantly associated with a greater probability of an Lp(a) change ≥ 20 mg/dL over time. In contrast to our results, they also reported associations between comorbidities such as diabetes and hypertension and changes in Lp(a)—findings that contrast with our results, where we did not observe such associations. The differences in the specific features of the studied populations might account for this discrepancy. Changes in Lp(a) levels over time were also reported in the large ARIC study, with a median follow-up of 15 years, where frequent reclassification occurred: many participants shifted from borderline-high (30–49 mg/dL) to high-risk categories (≥50 mg/dL) [22]. In addition, large fluctuations were observed among individuals randomized to placebo in the IONIS-APO(a) Rx and IONIS-APO(a)-LRx antisense oligonucleotide clinical trials [23]. In a population-based cohort of 11,669 patients, borderline Lp(a) levels often crossed diagnostic thresholds over time—in 51% of those initially borderline—and nearly 25% had an intra-individual change ≥ 10 mg/dL; predictors of greater variability included female sex, atherosclerotic cardiovascular disease (CVD) history, statin therapy, and LDL-C ≥ 100 mg/dL [24]. Even among healthy adults, month-to-month measurements revealed wide ranges in Lp(a), with individuals showing differences from 14 to 229 mg/dL over a year [25]. Our findings are consistent with recent data from a large Korean multicenter study, which reported a median intra-individual variability of 26.3% and identified nearly 20% of participants as having high variability (>25% or >10 mg/dL) [16]. Importantly, variability was particularly pronounced among patients in the intermediate ‘gray-zone,’ leading to frequent risk reclassification. Moreover, high variability was associated with unfavorable biochemical profiles, including higher glucose levels, elevated inflammatory markers, and use of antihypertensive medications, further underscoring the potential clinical significance of temporal changes in Lp(a). These results reinforce our observation that a single lifetime measurement may not be sufficient for accurate cardiovascular risk assessment, particularly in borderline-risk individuals, and support the role of serial testing in refining risk stratification.

Another study of 40 adult patients with acute coronary syndrome (ACS) undergoing percutaneous coronary intervention found that Lp(a) concentrations increased three months after the ACS event, once the patients’ condition stabilized. This finding suggests that ACS may represent another non-genetic factor influencing Lp(a) concentrations, and that measurements obtained during an ACS event may not accurately reflect an individual’s lifetime Lp(a) level [26]. Evidence of biological fluctuations has also been observed in healthy fertile women, in whom monthly measurements over one year revealed intra-individual variability ranging from 4% to 20%, with some exceeding 15% and rare cases approaching 50% of the mean value [27]. More recently, in a lipid clinic cohort, approximately one-third of patients showed a ≥25% change in Lp(a) concentrations despite clinical stability. Men showed slightly greater variability than women. Among hypervariable patients, changes in Lp(a) categories were observed in both directions, with some participants showing increases and others decreases; all women with hypervariable levels were postmenopausal [28]. Other studies have also implicated hormonal influences (particularly during menopause), as well as metabolic, inflammatory, and renal factors as potential contributors to Lp(a) fluctuations [29,30,31].

Our finding that individuals with a history of migraine demonstrated greater temporal variability in Lp(a) levels may reflect biologically plausible mechanisms linking migraine to fluctuations in vascular and inflammatory homeostasis. Migraine is increasingly recognized as a neurovascular disorder characterized by episodic endothelial dysfunction, transient inflammatory activation (including interleukin-6 and CRP surges), oxidative stress, and—in many patients—hormonal fluctuations. Each of these processes has been shown to influence hepatic lipoprotein metabolism or LPA gene expression, providing a mechanistic basis for modest short-term variability in Lp(a) [32,33]. The consistency of findings across different cohorts indicates that Lp(a) variability is at least partly of biological origin. Nevertheless, the overall evidence suggests that Lp(a) variability is a reproducible phenomenon that cannot be attributed solely to comorbidities, as also demonstrated in our study, where no statistically significant differences in the degree of Lp(a) variability were observed across most comorbid conditions, with the exception of stroke, where a trend toward greater variability was evident.

Potential contributions of pre-analytical, analytical, and post-analytical errors to the present results cannot be ruled out. Although every effort was made to minimize these errors, they could still have occurred in some instances.

From a clinical standpoint, Lp(a) variability is highly relevant. A single lifetime measurement may misclassify cardiovascular risk, either underestimating or overestimating a patient’s true long-term profile. The emergence of novel Lp(a)-lowering therapies further underscores the need to understand temporal variability, as treatment initiation and monitoring strategies may rely on accurate and reproducible measurements [34,35].

In summary, growing evidence from our study and others challenges the assumption of lifelong stability of Lp(a). While genetic determinants remain the dominant factor in defining Lp(a) levels, biological and methodological variability may result in clinically relevant reclassification. Future research should establish evidence-based recommendations on when repeat measurements of Lp(a) are warranted and in which populations they are most informative. From a clinical perspective, reliance on a single lifetime measurement of Lp(a) may lead to both underestimation and overestimation of cardiovascular risk. Our findings underscore the potential clinical impact of Lp(a) variability and highlight the need for further research to define which patient groups would benefit most from repeat testing. Although a proportion of observed changes falls within expected analytical variability, we emphasize that the purpose of this study was to quantify real-world intra-individual variation. Our findings highlight that even in a stable outpatient population, Lp(a) levels fluctuate to a greater extent than traditionally assumed. While some shifts remain within laboratory error, variability ≥30% and ≥50% is unlikely to be explained solely by analytical noise and may represent true biological changes. These findings are important for clinicians interpreting serial Lp(a) results. At a population level, Lp(a) is relatively stable; however, intra-individual fluctuations observed in our cohort indicate that clinicians should apply Lp(a) results in the broader clinical context. We suggest the following pragmatic approach: repeat measurement may be considered when the initial value is near a therapeutic/diagnostic threshold (e.g., borderline values), before initiating targeted Lp(a)-lowering strategies or after a major clinical event; for patients with significant observed changes (≥30%), clinicians should reassess risk and consider confirmatory testing. This strategy balances assay reproducibility with the need to avoid misclassification of patients near cut-offs.

## 5. Conclusions

Our findings challenge the widely accepted assumption that Lp(a) concentrations remain fully stable over time. A significant change in Lp(a) levels was observed in the midterm follow-up. The current expert recommendation to measure Lp(a) at least once in a lifetime appears to be inaccurate and should be revised.

## 6. Limitations

First, although both Lp(a) measurements were performed in the same laboratory using the same immunoenzymatic assay, part of the observed intra-individual variability may reflect analytical imprecision. Reported inter-assay CV values for Lp(a) are <6%. To minimise the risk of misinterpretation, we presented variability across multiple thresholds, recognising that larger observed changes are unlikely to arise solely from assay performance.

Second, detailed information on changes in concomitant medications—including initiation or discontinuation of statins, other lipid-lowering agents, or anticoagulants—was not systematically collected. Although these therapies are not expected to materially influence Lp(a) levels, the absence of such data limits our ability to fully rule out their contribution.

Third, information on menopausal status, hormone therapy, and other hormonal influences was unavailable, which may be relevant given their potential effects on lipid metabolism.

## Figures and Tables

**Table 1:** Characteristics of the study group according to the level of Lp(a) concentration change between the baseline and follow-up measurement.

Variable		Lp(a) Change < 20% *n* = 703	Lp(a) Change ≥ 20% *n* = 560	*p*	Total N = 1263
		N (%)			N (%)
Sex	Men	218 (31.0)	176 (31.5)	0.857	394 (31.2)
	Women	486 (69.0)	384 (68.5)		869 (68.8)
Age	<40	17 (2.4)	18 (3.2)	0.376	35 (2.8)
	40–64	210 (29.9)	182 (32.5)		392 (31.0)
	65 and over	476 (67.7)	360 (64.3)		836 (66.2)
Hypertension		461 (65.6)	360 (64.3)	0.633	821 (65.0)
Hyperlipidemia		147 (20.9)	114 (20.4)	0.809	261 (20.7)
Diabetes		134 (19.1)	116 (20.7)	0.464	250 (19.8)
Coronary artery disease		65 (9.3)	65 (11.6)	0.170	130 (10.3)
Cerebrovascular disease		40 (5.7)	54 (9.6)	0.008	94 (7.4)
Asthma		54 (7.7)	49 (8.8)	0.718	103 (8.2)
Rheumatoid arthritis		42 (6.0)	30 (5.4)	0.826	72 (5.7)
Migraine		44 (6.3)	53 (9.5)	0.092	97 (7.7)
Thrombophlebitis		15 (2.1)	13 (2.3)	0.822	28 (2.2)
Myocardial infarction		24 (3.4)	16 (2.8)	0.575	40 (3.2)
Smoking		65 (9.2)	63 (11.3)	0.157	128 (10.1)
**Variable**		**Me (Q1–Q3)**		***p***	**Me (Q1–Q3)**
Body mass index, kg/m^2^		27.5 (24.5–30.9)	27.5 (24.8–31.1)	0.707	27.5 (24.8–30.9)
Total cholesterol, mg/dL		178.5 (151.0–210.0)	180.0 (148.0–210.0)	0.452	179.0 (149.0–210.0)
LDL cholesterol, mg/dL		100.0 (75.00–131.00)	99.5 (70.0–128.0)	0.253	100.0 (73.0–130.0)
HDL cholesterol, mg/dL		54.0 (45.0–64.0)	53.0 (45.0–63.0)	0.168	54.0 (45.0–64.0)
Triglycerides, mg/dL		106.0 (81.0–140.0)	107.0 (82.0–151.0)	0.094	107.0 (81.0–144.0)
Hemoglobin A1c, %		5.6 (5.3–5.9)	5.6 (5.3–5.9)	0.625	5.6 (5.3–5.9)
C-reactive protein		2.0 (1.0–3.0)	1.6 (1.0–3.0)	0.870	2.0 (1.0–3.0)
Homocysteine, µmol/L		12.0 (10.1–14.6)	12.1 (9.6–14.4)	0.355	12.0 (9.9–14.6)

**Table 2:** Baseline and follow-up Lp(a) measurements results.

Parameter	Mean	Standard Deviation	Median	Quartile 1	Quartile 3
Lp(a) 1st measurement (mg/dL)	21.7	30.7	9.0	4.0	23.0
Lp(a) 2nd measurement (mg/dL)	21.9	30.6	8.8	4.9	24.0
The change between 1st and 2nd measurements (mg/dL)	4.5	8.8	1.8	0.6	4.7
The change between 1st and 2nd measurements (%)	36.2	288.0	16.7	6.5	33.3

## Data Availability

Data available on request from corresponding authors due to strict privacy and confidentiality regulations.
